# Improving physical activity, pain and function in patients waiting for hip and knee arthroplasty by combining targeted exercise training with behaviour change counselling: study protocol for a randomised controlled trial

**DOI:** 10.1186/s13063-018-2808-z

**Published:** 2018-08-07

**Authors:** Jane O’Brien, Kyra Hamilton, Andrew Williams, James Fell, Jonathan Mulford, Michael Cheney, Sam Wu, Marie-Louise Bird

**Affiliations:** 1 School of Health Sciences, University of Tasmania, Launceston, Australia; 2 School of Applied Psychology, Menzies Health Institute Queensland, Griffith University, Brisbane, Australia; 3 Sports & Exercise Science, School of Health Sciences, University of Tasmania, Launceston, Australia; 4 Launceston General Hospital, Launceston, Australia; 5 Department of Health and Medical Sciences, Swinburne University of Technology, Melbourne, Australia; 6 Department of Physical Therapy, Faculty of Medicine, University of British Columbia, British Columbia, Canada

**Keywords:** Osteoarthritis, Physical activity and Health Action Process Approach

## Abstract

**Background:**

Osteoarthritis often results in prolonged periods of reduced physical activity and is associated with adverse health outcomes, including increased risk of cardiovascular and metabolic diseases. Exercise interventions for patients on the waiting list for arthroplasty can reduce the risk of long-term adverse outcomes by increasing activity levels. However, uptake and ongoing positive rates of physical activity in this population are low and the impact of pre-operative behaviour counselling on exercise is not known.

**Method/design:**

The exercise and behaviour change counselling (ENHANCE) trial is a two-arm assessor-blind randomised controlled trial to assess the effectiveness of a 12-week exercise intervention designed to improve long-term physical activity and functional abilities for people awaiting arthroplasty. Participants on the waiting list for hip and knee arthroplasty are recruited from one clinical site in Australia. After collection of baseline data, participants are randomised to either an intervention or control group. The control group receive usual care, as recommended by evidence-based guidelines. The intervention group receive an individualised programme of exercises and counselling sessions. The 12-week exercise programme integrates multiple elements, including up to five in-person counselling sessions, supported by written materials. Participants are encouraged to seek social support among their friends and self-monitor their physical activity. The primary outcome is physical activity (daily step count and percentage of day spent in sedentary activities). Secondary outcomes include pain ratings, physical function, psychosocial factors and changes in clinical markers linked with potential common chronic diseases (diabetes and cardiovascular disease). All outcomes are assessed at baseline and 26 weeks later and again at 26 weeks post-surgery.

**Discussion:**

This study seeks to address a significant gap in current osteoarthritis management practice by providing evidence for the effectiveness of an exercise programme combined with behaviour counselling for adults waiting for hip and knee arthroplasty. Theory-driven evidence-based strategies that can improve an individual’s exercise self-efficacy and self-management capacity could have a significant impact on the development of secondary chronic disease in this population. Information gained from this study will contribute to the evidence base on the management of adults waiting for hip and knee arthroplasty.

**Trial registration:**

Australian New Zealand Clinical Trials Registry, ACTRN12617000357358. Registered on 8 March 2017.

**Electronic supplementary material:**

The online version of this article (10.1186/s13063-018-2808-z) contains supplementary material, which is available to authorized users.

## Background

Osteoarthritis (OA) is a common musculoskeletal problem, which affects over 40% of adults over the age of 70 years [1] and is associated with substantial pain and disability [2]. Patients with advanced OA of the hip or knee are often referred for surgery to replace the affected joints. Internationally, the waiting time for surgery varies from 6 to 12 months [3]. Due to pain, physical activity levels in patients with OA are substantially lower than levels in non-arthritic older people [4]. The prolonged periods of reduced physical activity during the wait for elective surgery significantly increase the risk of developing other major health problems, such as cardiovascular and metabolic diseases [5]. Inactivity may exacerbate physical impairments, such as muscle weakness, resulting in an increased rate of functional decline [6] and reduced rate of recovery post-surgery. In contrast, substantial research supports the benefits of regular physical activity in the prevention and treatment of chronic medical conditions including OA [7, 8] at all stages of the disease.

Previous research has largely concentrated on the effects of pre-operative exercise on post-operative pain and mobility [9]. Despite small improvements of 10% in physical activity after joint replacement, activity remains much lower than in healthy people [10]. The reason for this is unknown but may be because patients’ adherence to exercise tends to drop over time [11] and clinicians often do not appreciate and promote exercise as a treatment option [12]. Consequently, the immediate small positive benefits in pain and mobility are not sustained, as participants are not adequately supported to maintain exercise levels once the intervention finishes. An individualised intervention programme with a counselling component may address the challenge of achieving prolonged increases in physical activity for patients with OA.

Physical activity interventions based on social psychological theory are more effective than atheoretical ones in promoting health-protective behaviour [13]. In addition, interventions are more cost-effective when they have theoretically devised approaches and clearly measurable constructs. A theoretical basis for the intervention components and inclusion of key psychological measures based on theory can demonstrate *how* the intervention works, highlighting the processes by which behaviour change occurs [14]. The Health Action Process Approach (HAPA) [15] stipulates a dual-phase approach to action: a *motivational* phase (i.e. outcome expectancy, risk perception, action self-efficacy and intention) and a *volitional* phase (i.e. maintenance self-efficacy, recovery self-efficacy, action planning, coping planning and action control). According to HAPA, intention is a necessary but insufficient condition for action initiation and persistence. People also need to identify effective volitional self-regulatory strategies to enact their intentions and prevent relapse. The effectiveness of HAPA-based interventions has been demonstrated in multiple populations and contexts and across multiple health behaviours [15–21]. The use of this framework to promote physical activity in people with OA does not appear in the literature. However, HAPA has recently been used to identify six physical activity attributes that are most salient to adults with knee OA [22], which supports this framework in this population.

This report describes the methodology of the ENHANCE trial, a randomised controlled trial to test the effectiveness of the ENHANCE programme for a usual care control group. This study seeks to test the hypothesis that the combination of a targeted exercise prescription with behaviour change counselling based on HAPA will be more effective than usual care in increasing the number of daily steps at all-time points after intervention. Concurrently less pain, improved function and reduced metabolic and cardiovascular risks factors will be recorded.

## Methods/design

**Fig. 1:**
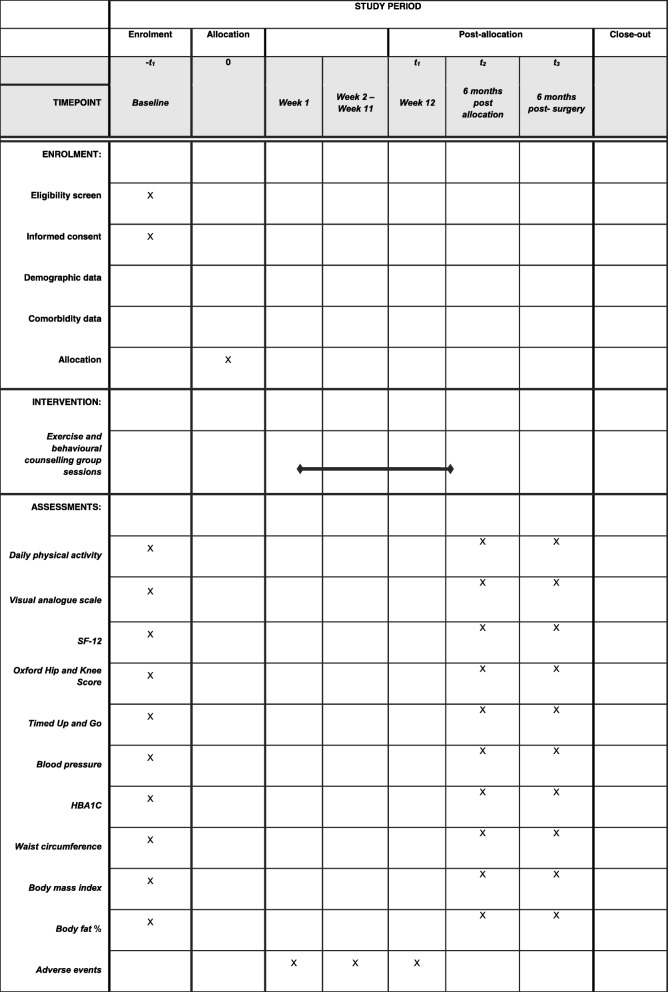
SPIRIT figure of the ENHANCE study protocol. SF-12 Short Form 12-item health survey on medical outcomes

The ENHANCE study is an open two-arm parallel-group randomised controlled trial. This is a superiority trial to determine if there is a clinically relevant difference between exercise and behaviour change counselling versus usual care. The 12-week ENHANCE programme is delivered to trial participants by an accredited exercise physiologist (AEP) in a university exercise physiology clinic. Minimum training at this level is a bachelor’s degree in sports science (kinesiology) and a professional honours qualification (4.5 years total full-time study at a tertiary institution). Data are collected at baseline and 26 weeks, and at 26 weeks post-surgery. A schematic diagram of enrolment is shown in Fig. 1.

### Setting

The study is conducted in Australia at Launceston, Tasmania. The ENHANCE programme is delivered in an exercise physiology clinic at a major university.

### Inclusion criteria

To be eligible to participate in the trial, patients have to meet the following criteria: new and existing patients on the surgery waiting list (<6 months on list), aged 80 years and younger (due to older people being less mobile and more sedentary and having more comorbidities and poorer cognition), able to read and understand English, willing to participate in a 12-week exercise intervention and provide informed consent.

### Exclusion criteria

Patients will be ineligible to participate in the trial if they meet one or more of the following criteria: an unstable medical condition whereby participation in exercise may present an additional health risk as determined by the consulting surgeon, prior diagnosis of a progressive neurological condition (e.g., Parkinson’s disease) or confined to a wheelchair.

Eligible participants will be invited by a nurse researcher attached to the Launceston General Hospital Orthopaedic Services to participate in the study. They will be provided with an information package and consent form. Potential participants will be provided with further information by a researcher who is an AEP. The AEP will also conduct additional clinically relevant screening of potential participants for conditions that could be made worse by exercise. Participants do not receive any incentives.

### Development of the ENHANCE programme

The development of the ENHANCE programme has followed a two-stage process and is informed by the HAPA. First, international guidelines on the development of exercise interventions for adults waiting for hip or knee surgery were reviewed. These guidelines recommend that exercise interventions for people with OA should be multi-dimensional and include exercise to improve whole-body muscle strength, mobility and aerobic capacity. It was, therefore, determined that including participants with both hip and knee arthroplasty widens the applicability of this trial, especially as the current activity levels for both cohorts are similar following arthroplasty.

Second, a series of interviews with people with OA and experienced health-care professionals was conducted. A draft intervention was then produced, which was refined by expert consensus.

### Description of the intervention

The ENHANCE programme is a 12-week programme consisting of 24 group classes of progressive exercise intervention combined with five group counselling sessions, delivered after the exercise class. Groups will commence and continue as discrete groups with a maximum of five participants per group. The classes are run twice a week for 60 min each. All sessions are delivered by an AEP. In accordance with recent recommendations for chronic disease management and physical activity guidelines, the intervention is stage-adapted and participants are encouraged to exercise within their own capabilities, which depend on individual symptomatology. Participants in the intervention group also receive an ENHANCE workbook, which includes printed material tailored for people with OA and informed by the principles of HAPA. The workbook was developed by the research team (Additional file 1) and includes general information about the benefits of exercise, setting goals, forming habits and making plans. The booklet describes various activities, which are either scheduled as part of the behaviour counselling face-to-face sessions or are reflective activities for participants to complete at home.

#### Exercise programme

The exercise programme will be individualised based on the assessment at entry by an AEP who has experience in the delivery of exercise to people with chronic health conditions. The intensity and progression of exercises will be determined on an as-needs basis and will include both the class-based programme and home-based exercises. The required elements of the exercise programme will be based on clinical evidence for hip and knee OA [23] and are outlined in Table 1. The dose will be determined by the supervising AEP in negotiation with each participant’s preferences and clinical status.

**Table 1:** Exercise components

Exercise component	Supervised (twice weekly)	Unsupervised (home)
Aerobic component	40–60% HR max, 12–14 repetitions; walking, cycling or arm ergometry	Low to moderate intensity (40–60% HR max, 12–14 repetitions); a home programme aiming to accumulate 20–30 min a day for 2–5 days a week will be progressively introduced where safe and practical
Flexibility: static stretching	Building to 3–5 stretches of up to 30 s duration	Initially, once daily at home to comfortable end of range (up to 15 s holds)
Strengthening isometric exercises	40% MVC with up to 10 holds for up to 6 s	Where safe and practical, this will be expanded to the home exercise programme and repeated daily
Strengthening isotonic exercises with TheraBands	40% MVC up to 15 repetitions	Will commence at low levels (40% MVC with between 10 and 15 repetitions) and progress when possible to higher loads and fewer repetitions to stimulate strength gains; once weekly

*HR max* maximum heart rate, *MVC* maximal voluntary contraction

The AEP will regularly review participants’ original or amended goals, participation in their home exercise programme since the previous class, barriers experienced, motivation for exercise, recent symptoms and pain status. Attendance and adverse events will be documented.

#### Behaviour change counselling programme

Behaviour change strategies and the principles of the counselling component of the intervention are guided by HAPA. The principles are designed to increase self-efficacy for exercise and management of other OA-related symptoms as well as improving the ongoing participation in positive health behaviours. Table 2 presents the content of the five behaviour counselling sessions, which are delivered as exercise coaching group sessions. Each session has a certain topic, matched with the mode of consultation. Face-to-face counselling sessions deal with the topics ‘Why is exercise good for me?’, ‘Building confidence’, ‘Creating plans and habits’, ‘Staying in control’ and ‘Visualising the future’. Behaviour change methods emphasised in this intervention include self-monitoring, goal setting, positive reinforcement, positive self-talk, self-reward, motivational advice and relapse prevention coaching. Over the 12 weeks, the HAPA-based intervention will comprise key components that map behaviour change techniques onto specific HAPA psychological constructs, drawn from theoretical and empirical evidence. Table 3 provides the theoretical foundation for the proposed HAPA-based intervention, outlining key components, potential behaviour change techniques and potential mediators.

**Table 2:** Overview of the behavioural counselling components of the ENHANCE intervention

Session	1	2	3	4	5
Intervention week	1	2	3	6	12
Main topic	Why is exercise good for me?	Building confidence	Creating plans and habits	Staying in control	Visualising the future
Theoretical aspects	General health benefits of physical activity and recommendations for enhancing physical activity Goal setting for the end of the intervention Organisational aspects of the programme Begin exercising hints and safety tips	Previous success stories Sharing success stories	Goal setting, action planning and coping for the following week	Identification of individual barriers to and resources for physical activity (significance of social support) Strategies for dealing with barriers	Goal setting, action planning and coping for the future post-surgery

**Table 3:** Proposed HAPA-based intervention components, behaviour change techniques, and potential mediators

Intervention components	Behaviour change techniques	Potential mediators
Motivational component		
Providing information on the risk factors of a sedentary lifestyle	Information provision Mental imagery	Risk perception
Providing information on the benefits and advantages of regular walking	Information provision Mental imagery	Positive outcome expectancy
Establishing confidence to start regular walking	Identification of resources Modelling (modelling by others) Mental imagery Verbal persuasion	Action self-efficacy
Formulating the intentions of regular walking	Intention formation Goal setting	Intention
Volitional component		
Making plans on when, where, how and with whom to conduct regular walking	Planning exercise	Action planning
Developing strategies to cope with the barriers that may interfere with regular walking	Identification of barriers Problem-solving	Coping planning
Developing confidence in maintaining regular walking with barriers, as well as resuming regular walking if interrupted	Mental imagery Mastery experience (past experience)	Maintenance self- efficacy Recovery self-efficacy
Developing strategies to remind and monitor regular walking	Self-monitoring exercise Reminders and sign-in table	Action control

*HAPA* Health Action Process Approach

Participants work with the AEP to identify small, gradual changes to exercise patterns that can be mastered and maintained, rather than drastic changes that are less likely to be maintained over time. This facilitates a sense of mastery and confidence, which can be built upon throughout the intervention to build self-efficacy. Each intervention contact results in a behaviourally specific action plan that specifies exactly what the participant intends to do and when. Also in the contact sessions, barriers and supports are identified, confidence is assessed and problem-solving is discussed as necessary. These steps are repeated during intervention counselling sessions, with goals being adjusted as necessary. Participants are encouraged to set their own goals based on their fitness level and circumstances.

### Usual care

Participants in the control group receive a generic information brochure at their baseline assessment point, *Arthritis (osteoarthritis) and exercise*, produced by Exercise is Medicine Australia [24]. Otherwise, participants allocated to usual care receive their concomitant care while they are on the surgical waiting list, attending for ENHANCE assessments at 6 months after baseline and at 6 months after their surgery.

### Sample size

The sample size has been determined to be 50 patients in each group to detect an increase of 1200 (24%) daily steps (alpha 0.05, power 0.8) based on research indicating usual changes in step count after lower limb joint replacement [10]. The study that our sample size calculations are based upon experienced a low withdrawal rate of 4%. We have made a conservative estimate of a 10% withdrawal rate and, hence, will recruit 110 participants.

### Randomisation

After completing all baseline data collection measures, participants are randomised to either the intervention group or the control group. Participants in the intervention group receive evidence-based physical activity guidelines supervised by an AEP for the 12 weeks. In addition, those in the intervention group receive an instructor-developed exercise booklet, along with innovative counselling classes five times directly after their exercise session. Participants in the control group will receive an information sheet with general advice on the benefits of physical activity for adults with OA. Participants in either group may receive relevant concomitant care and interventions.

The randomisation schedule has been prepared by a researcher independent of the research project using a computer-generated random number table. To conceal randomisation, the independent researcher has prepared consecutively numbered, sealed, opaque envelopes, which are kept in a locked location. As ENHANCE is an exercise intervention, blinding of the participants and the researchers who conduct the exercise sessions is not possible. Outcome assessors will not be involved in the delivery of the intervention and will be blinded to group allocations.

### Assessment

All staff at the trial site have been trained in the study protocol and the procedures for collecting informed consent and assessment data. A data collection training manual has been developed to guide research staff in the administration of the informed consent and data collection procedures. Assessments are completed in person at baseline, at 26 weeks and at 26 weeks post-surgery (Fig. 1). Once the data have been entered into the local database, they will be transferred to the data manager for the trial, who will compare the hard copies with the database to check accuracy. The data manager will check all the primary outcome variables and a randomly chosen 20% of the other variables. If there are any errors in the primary outcomes, or more than 1% errors for other variables, all data will be checked. The database will not include the assigned treatments. These will be recorded in a separate database, so that the statistician analysing the data will remain blind to treatment allocation.

Potential participants complete a brief screening by telephone conducted by the research nurse. During the screen, an in-person baseline assessment is scheduled for eligible participants. Informed consent is obtained before the assessment. All questionnaires are administered by an interviewer. All baseline and follow-up assessments will be conducted at the intervention site.

All assessment sessions (baseline [time 1], week 26 [time 2] and 26 weeks post-surgery [time 3]) will include a measurement of the primary and secondary outcomes. An ENHANCE questionnaire on data related to the HAPA constructs and a range of dimensions related to health outcomes will also be administered (see Table 2). The baseline assessment will collect additional demographic information related to health, medical history and general health status.

### Primary outcome measure

The primary outcome measure is daily physical activity (daily step count and percentage of day spent in sedentary activities). Participants will wear an activity monitor (activePAL™) for seven consecutive days. Data from the device will be recorded and uploaded to a computer. These data will be averaged over the 7 days of activity to calculate a daily step count. The percentage of the day spent in sedentary activities will also be recorded. This measure will be taken upon entry to the study (test 1), and at approximately 6 months (test 2), and then at 6 months after surgery (test 3).

### Secondary outcomes

The secondary outcomes include assessment of pain, function, general quality of life, clinical markers and psychological HAPA-based constructs. Specifically, tools to gather these data include measures of pain (visual analogue scale), quality of life (Short Form 12-item health survey on medical outcomes or SF-12), function (Oxford Hip and Knee Score) and Timed Up and Go. Clinical markers of comorbidities, such as cardiovascular and metabolic disease, will be assessed at all time points. Clinical markers relating to cardiovascular health (blood pressure), metabolic health (blood glucose and HbA1c) and body composition (waist circumference, body mass index and body fat percentage) will also be recorded. Psychological HAPA-based constructs (see potential mediators in Table 3) will be measured on multi-item psychometric instruments developed using standardised guidelines and validated in previous studies [15–21] and adapted for use in the current study. All secondary measures will be taken upon entry to the study (test 1), and at approximately 6 months (test 2), and then 6 months after surgery (test 3).

### Assessment of pain, function, general quality of life and clinical markers

- Physical functional will be measured using the Timed Up and Go assessment [25]. Briefly, this test involves the client standing up from an armless chair and walking around a cone placed 2.44 m in front of the chair, returning to the chair and sitting down. It is a reliable and valid measure of physical function that is used widely in clinical practice [26].
- Quality of life will be evaluated using the SF-12. This survey includes 12 items which capture information on two subscales describing physical well-being (the physical component summary) and mental well-being (the mental component summary). It is validated and has been widely used to measure quality of life in a range of populations [27–29].
- The visual analogue scale is a unidimensional valid measure of pain intensity [30], which has been shown to be sensitive to clinical change in people with arthritis. The Oxford Hip and Knee Function scales are clinically useful patient self-report tools, originally designed to be administered to determine the efficacy of joint replacement [31]. They contain 12 questions related to the impact of their condition on daily functioning over the previous 4 weeks. The hip assessment tool is valid, reliable and responsive to change [32]. The Oxford Knee Scale has good internal validity [31].
- Three indicators of body composition (body mass index, waist circumference and percent body fat) will be measured. These measures are simple and easy to attain and have been widely used as indicators of cardiovascular disease [33] and mortality risk [34] in large-scale population studies. Body mass index will be calculated from height and body weight. Waist circumference will be measured at the narrowest point between the base of the ribcage and the iliac crest as per standard methods. Body fat percentage will be assessed with bio-impedance analysis scales (Tanita BC-1000; Tanita Corp; Tokyo, Japan), which have previously been shown to be a reliable and reproducible method for determining body composition [35].
- Brachial blood pressure will be measured with an aneroid sphygmomanometer by an experienced practitioner using standardised techniques after 5 minutes of seated rest.
- Blood measures of metabolic health [blood glucose and glycosylated haemoglobin (HbA1c)] will be measured from finger-prick blood samples using point of care analysers. Random blood glucose will be measured using a Hemocue Point of Care device (Hemocue Glucose 201; Hemocue AB; Angelholm, Sweden), a reliable and valid device for measuring blood glucose [36]. HbA1c will be measured with a DCA Vantage point of care analyser (Siemens Healthcare, Tarrytown, NY). The DCA Vantage analyser has previously been demonstrated to be a reliable and valid method for determining HbA1c [37].

### Assessment of psychological HAPA-based constructs

- *Regular exercise* will be assessed using a multi-item scale. ‘Think about the past week. In general, how often did you do regular exercise?’ Responses are rated from 1 (never) to 4 (very often). ‘Think about the past week. In general, to what extent did you do regular exercise?’ Responses are rated from 1 (never) to 4 (a large extent). In addition, an open format item ‘Think about the entire past week and count, how many days you did exercise (e.g., if you exercised for at least 20 minutes at a time every day then the response is 7)?’
- *Habit strength* is assessed using the four-item Self-Report Behavioural Automaticity Index [38]. A sample item is ‘Do you agree that doing regular exercise is something… I do automatically?’ Responses are rated from 1 (strongly disagree) to 7 (strongly agree).
- *Intention* is assessed with three items. A sample item is ‘In regards to doing regular exercise over the next week, do you agree that… I intend to exercise?’ Responses are rated from 1 (strongly disagree) to 7 (strongly agree).
- *Attitude* is assessed with three items. A sample item is ‘Doing regular exercise over the next week would be… unpleasant.’ Responses are rated on semantic differential scales from unpleasant to pleasant, bad to good, or harmful to beneficial.
- *Social influence* is assessed with four items. A sample item is ‘In regards to doing regular exercise over the next week, do you agree that… People give me support to ensure I regular exercise?’ Responses are rated from 1 (strongly disagree) to 7 (strongly agree).
- *Perceived Behavioural Control* is assessed with four items. A sample item is ‘In regards to doing regular exercise over the next week, do you agree that… I have complete control over whether I do regular exercise?’ Responses were rated from 1 (strongly disagree) to 7 (strongly agree).
- *Barrier self-efficacy* is assessed with five items. A sample item is ‘I am confident I can do regular exercise over the next week… even when I am in pain.’ Responses are rated from 1 (not at all confident) to 7 (definitely confident).
- *Action planning* is assessed with four items. A sample item is ‘In regards to doing regular exercise over the next week, do you agree that I have made a plan regarding… When to regularly exercise?’ Responses are rated from 1 (not at all true) to 7 (definitely true).
- *Action control* is assessed with three items. A sample item is ‘During the last week… I have often had my regular exercise intentions on my mind?’ Responses are rated from 1 (not at all true) to 7 (definitely true).

### Programme adherence

Participants randomised to the exercise intervention will be allocated to exercise classes based on their starting date and availability. Adherence is categorised as follows: participation in 75% or more of the supervised sessions is excellent, participation in 50–74% of sessions is good, participation in 25–49% of sessions is moderate and participation in less than 25% of the sessions is poor. Adherence to exercise during the exercise intervention will be measured via attendance sheets at group training sessions. After the exercise intervention, participation in the prescribed home exercise will be evaluated via self-report (questionnaire) collected at test 2 and test 3.

### Data analysis

Differences in primary and secondary outcomes between groups will be compared using an intention-to-treat analysis. Once a participant is randomised to a study group, they will be considered a trial participant and analysed according to their allocated group, regardless of missing data for follow-ups or the amount of intervention received. Bivariate relationships will be tested with Pearson or Spearman correlations, independent *t*-tests or Mann–Whitney *U* tests (to examine relationships between the dependent variables and the independent variables) or chi squared tests for categorical variables. Each primary and secondary outcome will be analysed using a two-way repeated-measures analysis of covariance (ANCOVA) to determine differences between groups (exercise versus usual care) and between time points (test 1, test 2 and test 3). In addition, effects of the intervention targeting the psychological HAPA-based constructs on regular exercise is tested along with the key processes (mediation) involved. Data will be analysed with a linear mixed model with condition as between-participants effects and time points as within-participants effects. A path analysis will be used to determine mediation effects. Data analysis will be performed using statistical software STATA (STATA 13, Stata Corp, College Station, TX, USA) and Mplus. Statistical significance is set at *p* < 0.05 for all analyses.

### Confidentiality

All data collected will be regarded as confidential and securely stored.

### Informed consent

Eligible participants are assessed for inclusion in the study. All participants are asked to provide written informed consent prior to enrolment in the study.

### Ethical and organisational review

Ethical approval for the ENHANCE trial has been granted by the Human Research Ethics Committee (H0016201) at the participating site. The protocol conforms to CONSORT guidelines [39] for reporting non-pharmacological interventions.

## Discussion

The ENHANCE trial is an intervention designed to improve physical activity participation and health-related outcomes in patients on the waiting list for hip or knee arthroplasty. This study is the first of its kind to use a supervised exercise intervention with behavioural counselling based on HAPA to motivate and change behaviours that support increases in physical activity. Findings from this study will add to the growing research literature on essential components for the optimum delivery of exercise for chronic disease management. Furthermore, this study may yield insights to our understanding of the strengths and limitations of a novel behaviour change intervention that is embedded in an exercise programme for people with a chronic disease, using OA as a model for chronic disease. An exercise intervention that can increase overall physical activity levels and improve individuals’ exercise self-regulatory and self-management capacity could be significant in improving the management in the community of people on the waiting list for hip and knee arthroplasty.

### Limitations

The authors acknowledge the following limitations: a single-centre trial, lack of a cost-effectiveness analysis, and limited patient and public involvement in the design of the intervention (Additional file 2).

### Trial status

The trial is currently ongoing.

## Additional files

Additional file 1: Participant booklet. (PDF 705 kb)

Additional file 2: SPIRIT 2013 Checklist. (DOC 138 kb)

## Abbreviations

AEP: Accredited exercise physiologist
ANCOVA: Analysis of covariance
ENHANCE: Exercise and behaviour change counselling
HAPA: Health Action Process Approach
HR max: Maximum heart rate
MVC: Maximal voluntary contraction
OA: Osteoarthritis
SF-12: Short Form 12-item health survey on medical outcomes
