# Supplementary material for: Improving physical activity, pain and function in patients waiting for hip and knee arthroplasty by combining targeted exercise training with behaviour change counselling: study protocol for a randomised controlled trial
Source: Trials. 2018 Aug 7;19:425. doi: 10.1186/s13063-018-2808-z (PMC6081939; doi:10.1186/s13063-018-2808-z)
Supplement: Supplementary file 1 — Participant booklet. (PDF 705 kb) [file 13063_2018_2808_MOESM1_ESM.pdf]

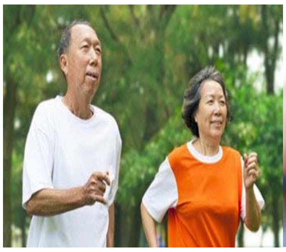 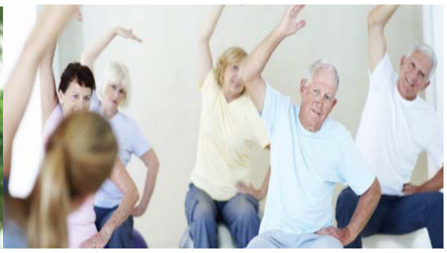 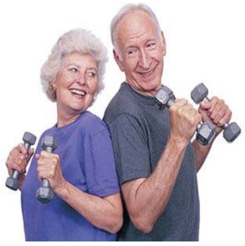

The ENHANCE (Exercise aNd beHaviour chANge CounsElling) program for people awaiting arthroplasty

PARTICIPANT BOOKLET

Brief outline of the program

The *ENHANCE* (Exercise aNd beHaviour chANge CounsElling) program for arthroplasty peoples is a program focused on building regular exercise and extra walking steps into ones daily routine.

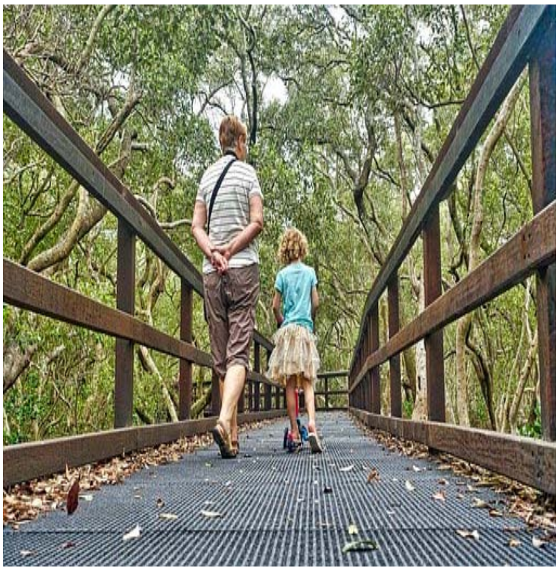

What is exercise?

Physical activity is any movement produced by muscles that requires energy. **Exercise** is **planned, structured, repetitive**, and aims to improve or maintain one or more components of physical fitness. Exercise is made up of the following components i) aerobic (e.g. walking, cycling or swimming), ii) flexibility (e.g. stretching, tai chi), iii) strengthening (e.g. using a theraband or lifting weights).

Therefore, whenever you see the word **"exercise"**, this applies to planned and structured physical activities performed with the aim of improving or maintaining your health and fitness.

What is regular exercise?

Regular exercise = performing exercise for **at least 20 minutes at a time on at least 2 days of the week**.

This program focuses on your current and future exercise behaviours. The aim of this program is to help you to begin or continue to exercise and, importantly, to incorporate additional walking steps into your daily routine. The information, tips, and strategies provided in this program are based on behavioural theory and scientific evidence and are designed to increase your level of exercise and for these to become part of your lifestyle.

Points to remember

- You may experience some discomfort in the affected joint during exercise — this is normal and does not indicate a worsening of the Osteoarthritis. However, a substantial increase in pain or swelling (that lasts more than several hours) during, or following, exercise can suggest that modifications to your exercise program are required. Your exercise physiologist in the supervised classes can assist you if this happens.
- You will gain the greatest benefits if you perform the exercises regularly. This booklet will provide strategies that you can use at home to help you incorporate regular exercise into your lifestyle into your daily routine.

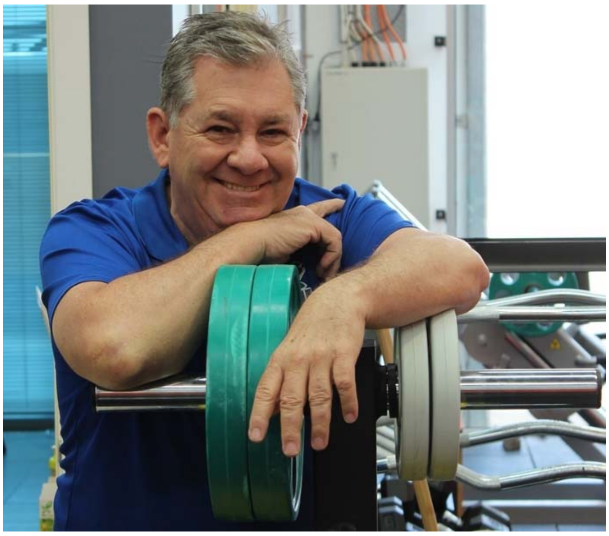

Overview of Program Structure

Session 1, Week 1: Why Is Exercise Good For Me Page 5

- Why is exercise good for me?
- What are the advantages/disadvantages about exercise?

Session 2, Week 2: Building Confidence Page 10

- What can I control doing exercise?
- What skills do I have to do exercise?

Session 3, Week 3: Creating Plans and Habits Page 18

- What are my exercise goals?
- How do I incorporate exercise into my lifestyle?

Session 4, Week 6: Staying in Control Page 28

- What can I still control doing exercise?
- What is working/not working for me to continue to exercise?

Session 5, Week 12: Visualising the Future Page 32

- What are my reflections on my exercise routine?
- What do I want my future and post-surgery exercise routine to be like?

SESSION 1 - WHY IS EXERCISE GOOD FOR ME?

Osteoarthritis is a chronic joint disease that causes pain, stiffness, swelling, joint instability and muscle weakness, all of which can lead to impaired function and reduced quality of life. Exercise is recommended by all clinical guidelines for the management of osteoarthritis (Bennell and Hinman, 2013).

Osteoarthritis is the most common type of arthritis and the major cause of chronic musculoskeletal pain and mobility disability in elderly populations worldwide. In 2007, 7.8% of Australians had osteoarthritis (Bennell and Hinman, 2013). The knees, followed by the hips, are the most commonly affected weight-bearing joints.

image removed

Role of exercise in treatment of hip and knee osteoarthritis:

Regular exercise can improve impairments associated with osteoarthritis including muscle strength, joint range of motion, proprioception (perception of movement and spatial orientation), balance and cardiovascular fitness (endurance) (Bennell and Hinman, 2013). Other potential benefits of exercise for people with osteoarthritis include improvements in mobility, falls risk, body weight, mental health and a range of chronic disease.

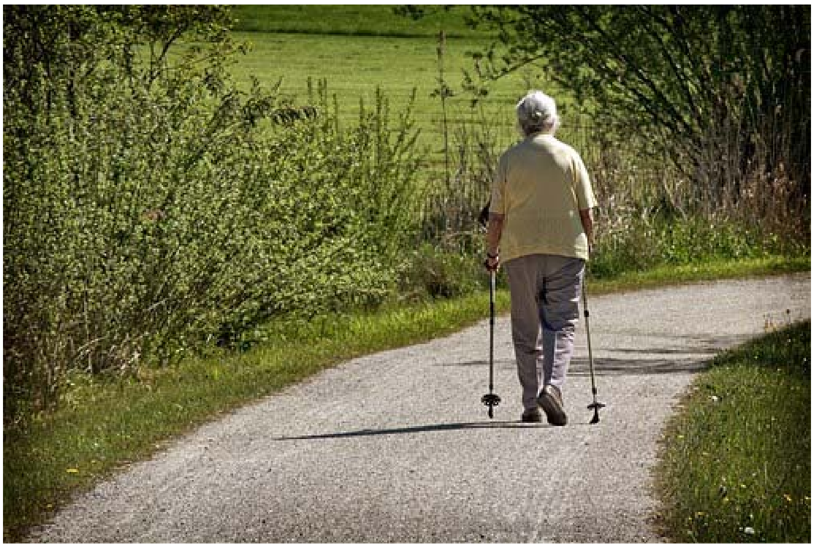

A SUCCESSFUL OUTCOME BEGINS BEFORE SURGERY!

While rehabilitation is essential in allowing a person to regain function and quality of life following a total joint replacement surgery, pre-surgical physical conditioning – prehabilitation, or "prehab" – can improve the healing process and help improve function following surgery.

Prehab is the process of enhancing the fitness level and functional capacity of people before surgery.

The individualised and targeted exercises you will be doing in your supervised exercise sessions in the weeks leading up to surgery will:

- Speed up your overall recovery and healing
- Strengthen the muscles that will support your new joint
- Reduce fatigue and muscle soreness
- Improve your circulation

How prehab helps

A person's level of physical fitness, muscular strength, endurance and joint range of motion have all been shown to affect outcomes after joint replacement surgery – particularly when it comes to minimising muscle wasting and stiffness and returning to daily activities.

Prehab can benefit people in many ways:

- **Improved Strength and independence**: Helps people to move independently and/or to be less reliant on assistance post-surgery.
- **Improved Body Awareness**: After surgery, people must re-learn everyday movements, like getting up from a chair. Prehab teaches people about better body awareness in preparation for such tasks.
- **Improved Fitness Level**: Enhances healing and recovery rates, improves motivation and commitment during rehabilitation.
- **Improved function**: As people report less pain, better quality of life and more confidence after prehab.

image removed

Exercise Brainstorm **What do I think about exercising?**

1. **Record and rate some of your thoughts (higher number, more important)**

**Exercise Advantages**	**Importance 1 - 10**	**Exercise Disadvantages**	**Importance 1 - 10**

2. Do the advantages/disadvantages outweigh each other?

____

____

Session 1: Homework Task

Over the next week identify what has 1) helped you to exercise and/or 2) stopped you from exercising?

**1. Exercise motivators**	**2. Exercise barriers**

Session 1: Take Home Points

Why is doing exercise important for me?

____

____

See you next week!

SESSION 2 – BUILDING CONFIDENCE

By performing daily exercise in the lead up to your replacement, you can boost your overall health, and speed up recovery.

It is important that people believe they have sufficient personal resources, are confident, and have ability to perform tasks. We are going to spend this session thinking about how to gain more confidence in doing exercise and identifying what skills you already have to do exercise.

Discoveries of Session 1 Homework Task

1. **Let's discuss these as a group. Please write down those factors that were important to you in the box below.**

1. Exercise motivators	2. Exercise barriers

Sharing Stories

* all images and transcripts removed for privacy*

Female, x years of age *with the following conditions:*

The following describes the benefits of exercise and **how exercise makes this woman feel in her current health situation**:

Previous exercise history

Current exercise

How did you keep exercising when you were in pain?

Encouraging statements for people with osteoarthritis about exercise

3. Now think about your own successful experience with exercise. Please write about this experience below. If you cannot recall an experience, perhaps one of the example stories resonates with you.

____

____

____

____

____

____

4. Now, think about a self-motivating phrase that you could recall when being tempted to not exercise. For example,

It is not easy but it is worth it

Write your own self-motivating phrase here:

____

____

____

____

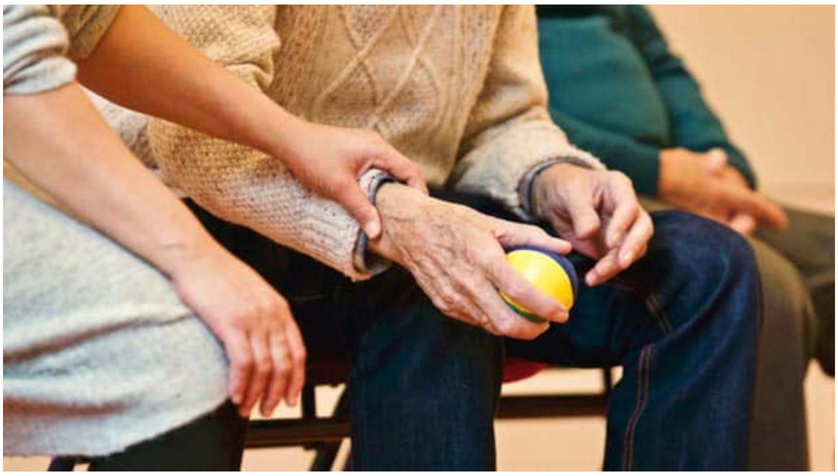

SESSION 2: HOMEWORK TASKS

Over the next week 1) document success stories for your exercising and 2) find cues that are consistent in your regular routines and environments which trigger you to exercise or think about exercising, e.g. family/friends/weather/a regular event.

1. Exercise Success	2. Exercise Cues

Session 2: Take Home Points

This week's exercise success for me is

____

____

See you next week!

SESSION 3 – CREATING PLANS AND HABITS

The next part of the program will help you to **build exercise (20-30min, 2-5 times per week) and extra walking steps into your regular routine**.

Research shows that developing habits is a good way to initiate and maintain regular exercise. Adding exercise habits into your everyday routines is a great way to keep active. You can develop a new exercise habit by doing a specific activity repeatedly at the same point in your routine. It will become automatic and easy to maintain.

Discoveries of Session 2 Homework Task

Share with the group one success and one cue that you recorded in your homework. Listen to other group members and write down any cues from the others that you want to try.

1 Exercise Success	2. Exercise Cues

Healthy Habits:

A habit is something you do **automatically**, like tying your shoelaces or brushing your teeth. Habits are formed when you do something **over and over** again in the **same place** or at the **same time**. We know that habits can affect our health in different ways, for example, driving to the shops instead of walking might make it more difficult to manage our weight. Getting into a routine and practising the healthy tips every day, helps turn them into healthy habits.

Ten Top Tips for Exercise:

The **Ten Top Tips** for exercise offers some tips for opportunities for small changes that people with osteoarthritis can make to stay active for longer.

The tips have been designed by a panel of exercise experts, and adults with osteoarthritis themselves. They are based on scientific evidence. The tips are designed to be easy to do, so that they become part of your everyday routine.

Some will get your heart pumping a little more and others will help you add extra walking steps into your normal routine. Try to do as many of them as you can. Remember, doing the tips at the same point in your normal routine will make it easier to turn them into habits.

Please read through the Ten Top Tips carefully. You will find a poster of these in your exercise folder. Choose 1 to do this week. Circle them here.

1<br>**Leave the house daily**<br>Ensure that you go out at least once per day. Going out reduces time spent inactive indoors.	2<br>**Make ad breaks active**<br>Stand up and walk around during breaks in TV programmes.
3<br>**Take a stand**<br>Stand up when waiting for a bus. Stay standing as long as possible.	4<br>**Time to stretch**<br>Set an alarm to go off every 20 minutes when inactive for long periods. When it rings get up and stretch.
5<br>**Rising and sinking**<br>When standing at a kitchen bench, increase your leg strength by standing on your tip toes and then slowly dropping back down to your heels.	6<br>**Watch your step**<br>Try to do at least 30 minutes of walking in total over the course of the day.
7<br>**Sit to stand with no hands**<br>Each time you stand up, try doing it without using your hands.	8<br>**Improve your posture**<br>A good posture will help your balance and reduce the likelihood of falling.
9<br>**Limber up**<br>Perform calf and chest stretches, knees up, finger walking, two-can lift and walk the line exercises in the same order each morning, at your own pace.	10<br>**Wall push-ups**<br>Do 10 push-ups against a wall each morning.

Adapted from: White I, Smith L, Aggio D, Shankar S, Begum S, Matei R, et al. On Your Feet to Earn Your Seat: pilot RCT of a theory-based sedentary behaviour reduction intervention for older adults. Pilot and Feasibility Studies. 2017;3(1):23.

Turning the tips into habits

- Plan ahead
  - How will you fit the tips into your daily routine?
  - Making a plan increases your chances of doing something
- Be prepared
  - Do you need to do anything differently (e.g. pack walking shoes)
- Repeat the tips at the same time or in the same place
  - This makes it more likely that you will form habits
- Keep track of your progress in the logbook

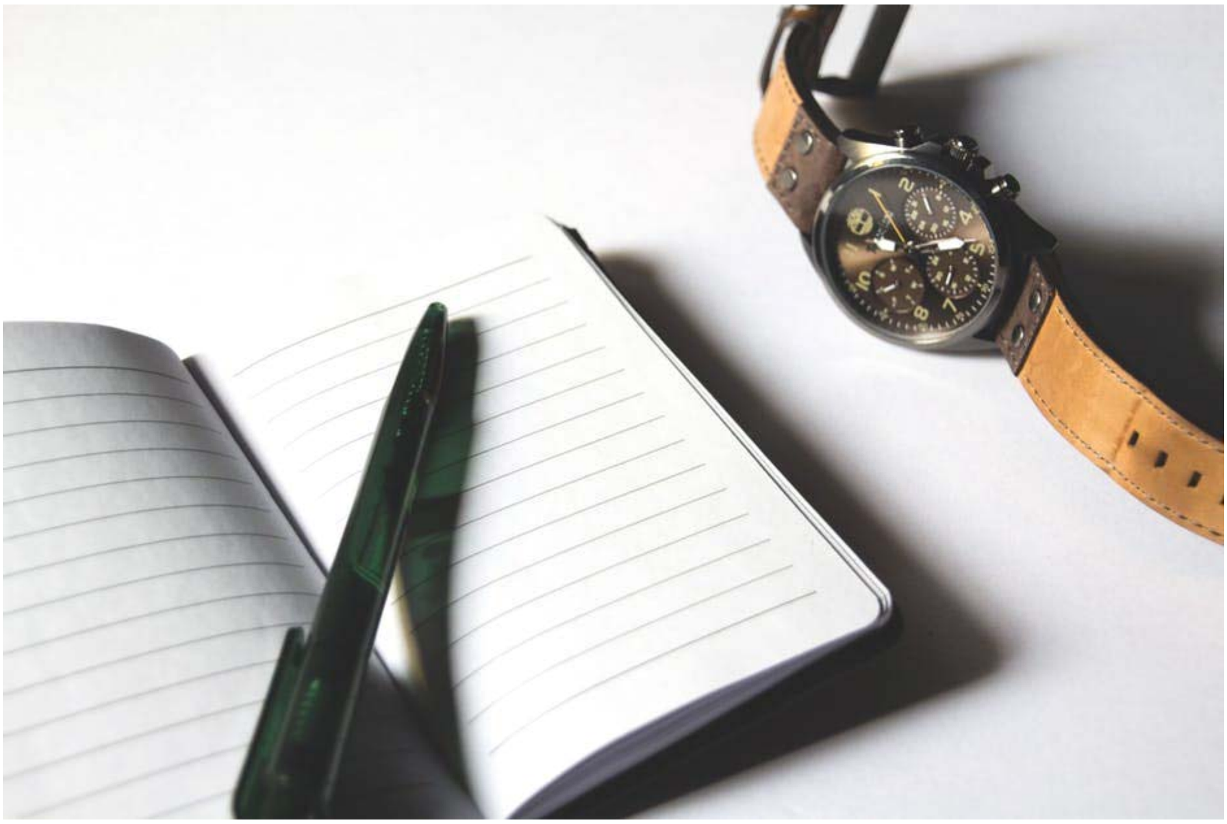

Incorporating the Ten Top Tips into your Routine.

Now make a plan!

Research shows that it is much more likely you will start to incorporate your chosen tips into your daily routine if you make a plan for **how, when and where** you are going to perform each of them throughout your week.

To start building your new habit, make a plan for each of your chosen tips below.

Here is an example:

Make a plan for **how, when and where** you are going to **add activity to an errand** at home.

How: I am going to stand up and walk around during breaks in the news

When: Every night

Where: At home during the evenings.

Summary: I will stand up and walk around during breaks in the news every night so I incorporate more walking in the evenings.

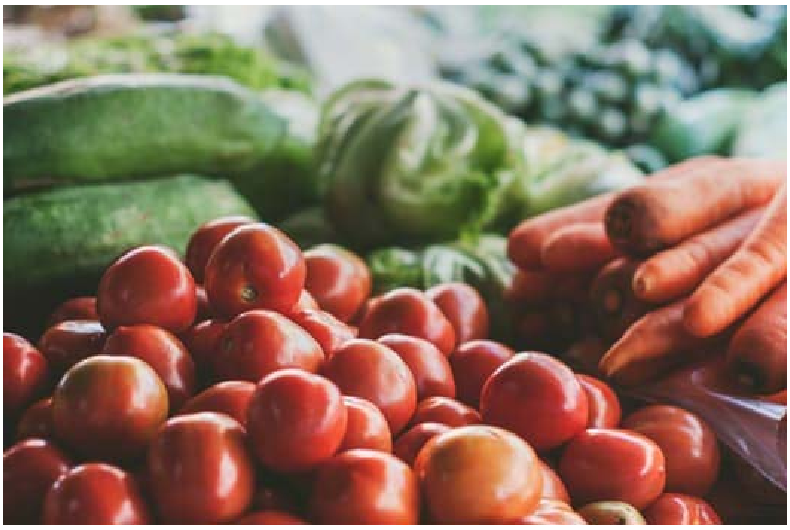

Make a plan for **how, when and where** you are going to do **One** at home.

How:

____

When:

____

Where:

____

Summary:

____

* image removed *

PLANNING

1 Exercise Tip	2. What or How	3. When	4. Where
Leave the house daily			
Make ad breaks active			
Take a stand			
Time to stretch			
Rising and sinking			
Watch your step			
Sit to stand with no hands			
Improve your posture			
Limber up			
Wall push-ups			

Get in the habit…

You will find it easier to form habits if you:

Plan ahead: Spend some time now thinking about how, when and where you will fit the tips into your current routines.

Start low, go slow: If a tip seems too hard, then build up to it gradually until you are comfortable doing it.

Keep going: Doing the activity repeatedly in your routine will make it easier to form a habit. If you forget to do one of your chosen tips, don't worry. Just keep going and do it next time as part of your routine.

Track your progress Keeping a record improves your chances of developing healthy habits. Use the tick sheet in the exercise folder each day to record whether you have done each of your chosen tips that day.

Session 3: Homework Tasks

Ten Top Tips tick sheet: Keeping track of your progress

Fill this tick sheet every day to record whether you managed your chosen tip(s). Keeping a record increases people's success in developing healthy habits. Below the Table, you can write details of how you are achieving the tips, and anything that particularly helps you use them. This information will help you plan for the next week.

RECORDING MY TIPS

Exercise Tip	Monday	Tuesday	Wednesday	Thursday	Friday	Sat	Sun
Leave the house daily							
Make ad breaks active							
Take a stand							
Time to stretch							
Rising and sinking							
Watch your step							
Sit to stand with no hands							
Improve your posture							
Limber up							
Wall push-ups							

What helps you to use the tips?

____

____

Session 3: Take Home Points

This week's habit success for me is

____

____

* image removed *

See you in Week 6!

SESSION 4 – STAYING IN CONTROL

The next part of the program will help you to stay in control of your exercise.

Research has shown that if an individual does not believe in their capability to perform a desired action, efforts to initiate or maintain the action may be difficult. Self-efficacy, however, can be different at different points in time in the decision making process. For example, you may have high confidence in your ability to exercise on a regular basis and take initiative (like we discussed in session 3) but have little confidence in your ability to maintain the desired behaviour or to recover from setbacks that stopped you exercising for a period of time. Adopting a new health behaviour might be much more difficult to adhere to than originally expected, but, having high self-efficacy to maintain the behaviour and to recover from setbacks you will invest more effort and persist longer, and when setbacks occur, will recover more quickly and maintain commitment to your goals.

Let us work more on building your self-confidence so you stay in control!

Discoveries of Session 3 Homework Task

Think about the plans you made in session 3, and reflect on the past few weeks following those plans, what helped you to stick to your plans?

____

____

What stopped you from sticking to those plans?

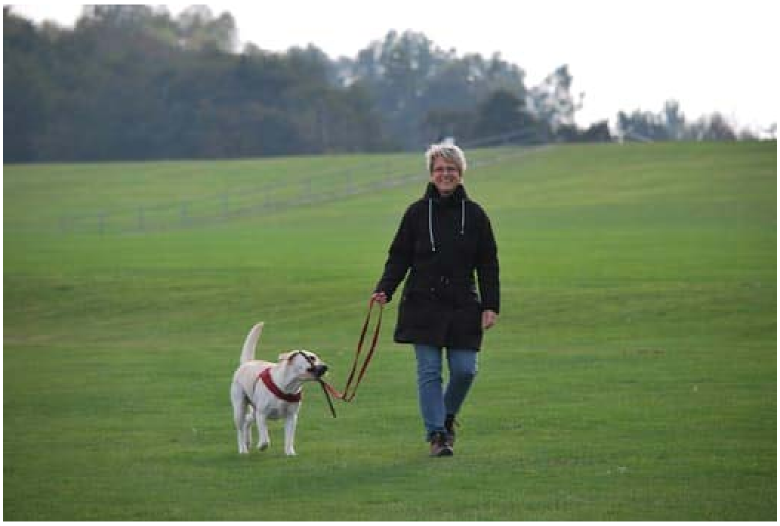

Now, reflect on that experience where you had a setback to exercising, and think about a successful experience where you were able to recover from the setback and start exercising again. Please write about this experience below.

Take some time to write down what the plan was that you made in Week 3.

____

____

____

Making plans to cope with set backs

Remember the critical situations you have faced in the last six weeks when you wanted to exercise but were unable to and some you might face in the future. Please note in the table below your possible critical situations and coping strategies you would use to cope with the help of the given example.

**If** I face difficult situations that might prevent me from being physically active… (which critical events)	…**then** I plan to overcome them by… (which strategies)
If *I experience pain*	***then*** *I will stop and rest a bit then keep going*

Session 4: Homework Tasks

Over the next few weeks follow your plans to maintain your behaviour and try to implement the strategies to overcome setback if you experience any.

Session 4: Take Home Points

This week's exercise coping strategy for me is

____

____

____

See you in Week 12!

SESSION 5 – REFLECTING ON THE PAST AND VISUALISING THE FUTURE

The next part of the program will help you to reflect on how you learned to build your confidence, create plans and stay in control. These are important strategies that you now have in your toolkit and you will be able to apply post-surgery.

Discoveries of Session 4 Homework Task

Share with the group one setback you faced and one coping strategy that you recorded in your homework. Listen to other group members and write down any coping strategies from the others that you want to try.

____

____

____

Now that you have reflected on the past, let's imagine your future!

Imagery is a technique used to enhance confidence, attention and concentration, and motivation. This will involve you mentally rehearsing or picturing your future actions in advance of doing the action. This will require you to draw on your experiences and imagine the steps required to participate in the action itself. This will also require you to imagine the feelings linked to performing the action and how you would feel after doing it.

We want to help you achieve your exercise goals post-surgery

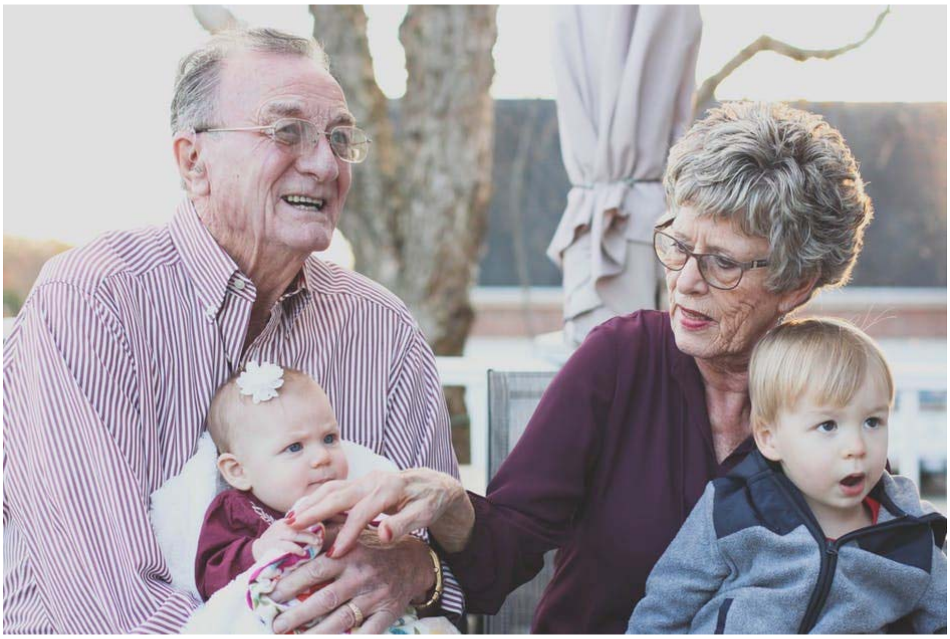

To help you do this we ask you to take 5 minutes of your time to complete this very simple mental exercise.

EXERCISE: You are more likely to carry out your physical activity goal if you make a decision about where and when you will do so and imagine doing it. Decide now when and how you will do physical activity post surgery, what activity or activities you plan on doing, and how you will go about doing so. You may find it useful do your activity just before or just after something else that you do regularly, such as going to work, eating dinner, or going for lunch.

Now close your eyes and visualize carefully and in great detail your exercise plans. Think of the things you will need to do: the equipment you will need, what you will need to bring with you, who you will need to tell, how you will get there, what the environment will look like, how you will be dressed, what it will feel like doing the activity, and how you will feel afterwards. It is important that that you actually see yourself doing your exercise. It is important that it is very detailed.

Now, write some brief notes on what you imagined on the lines below. This will help you remember what you imagined and help you achieve your goal.

____

____

____

____

Do you remember having to build up your confidence at the beginning of this program, it will be similar after you have had your surgery.

Being realistic about your expectations post-surgery is important.

Remember to start small with the recommendation of your surgeon and support of an exercise professional such as a Physiotherapist or Accredited Exercise Physiologist.

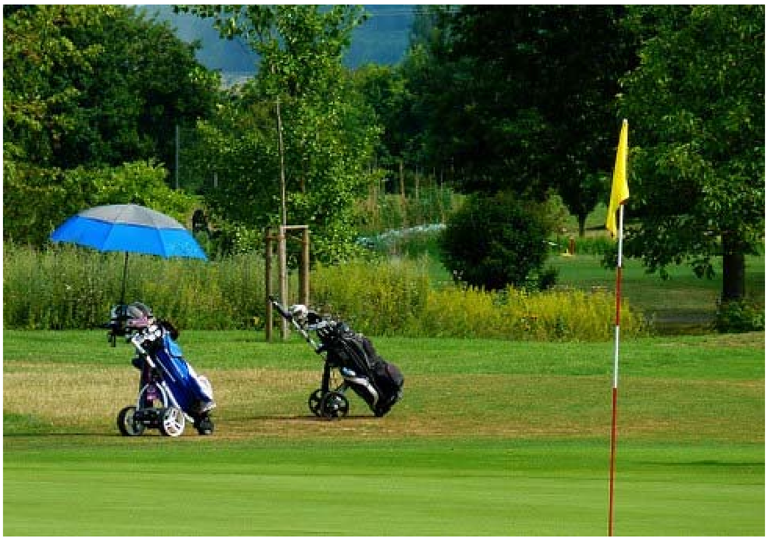

Session 5: Take Home Points

As a group, share in a group discussion what each of you can imagine doing after having your joint replacement.

After your surgery, you can use the skills that you have developed to realise this.

Overall summary

What do you now know about yourself and your capacity to exercise?

- Exercising for me is…

- What cues to exercise work really well for me…

- What tips am I going to try and implement…

- If I experience a set back then my plan is to….

---

* image removed *

Exercise Diary – please place a TICK ✓ to keep track of your progress

WEEK

	**Attended class**	**Completed home exercise program**	**Went for a walk**	**Participated in chosen tip**	**Pain scale (see attached page)**
**Monday**					
**Tuesday**					
**Wednesday**					
**Thursday**					
**Friday**					
**Saturday**					
**Sunday**					

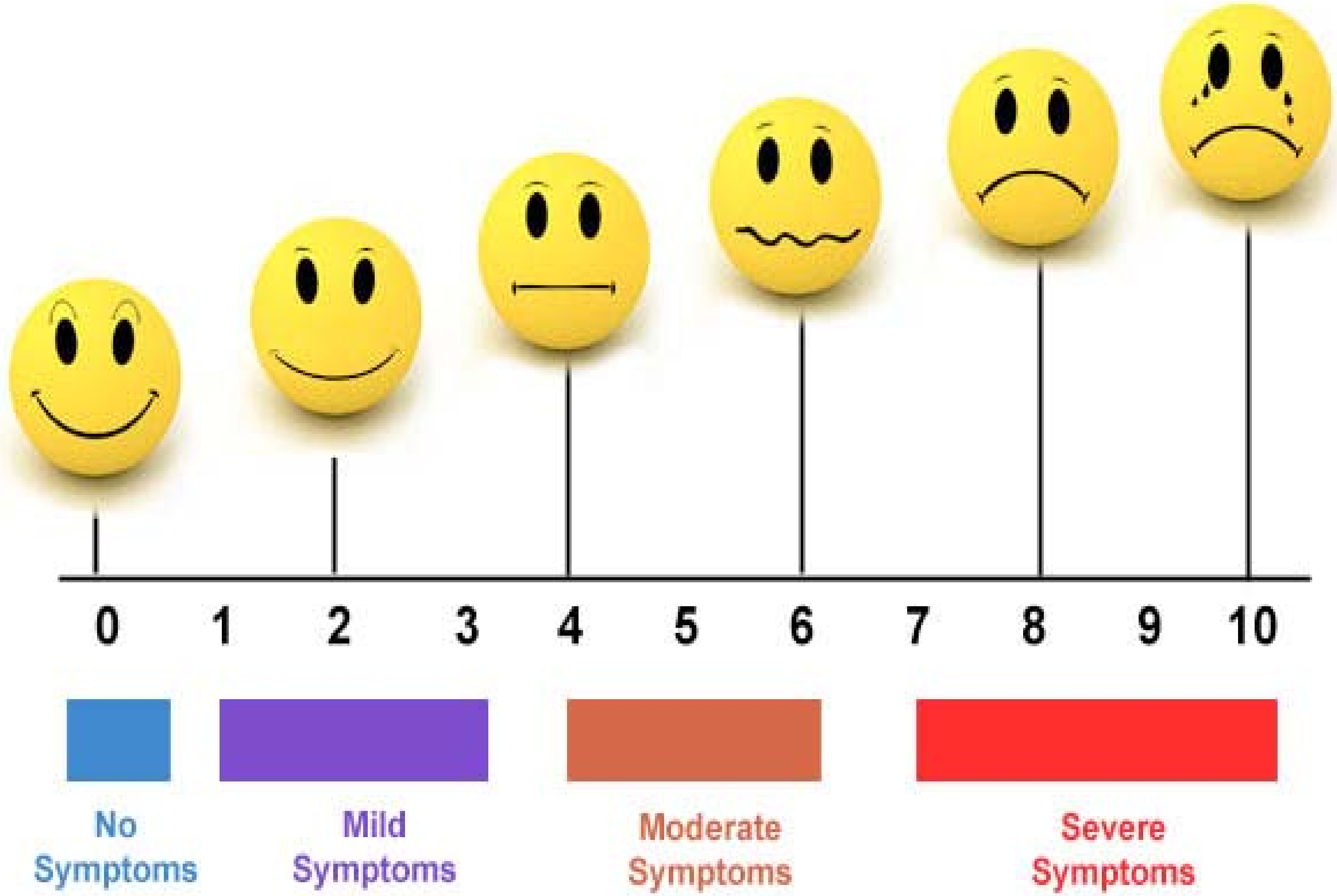
0
1
2
3
4
5
6
7
8
9
10
No Symptoms
Mild Symptoms
Moderate Symptoms
Severe Symptoms
